# Validation of a Real-Time PCR for the Diagnosis of *Leishmania* Species Using the *Hsp20* Gene

**DOI:** 10.3390/tropicalmed10050121

**Published:** 2025-05-01

**Authors:** Mayra Maldonado-Aroni, Nyshon Rojas-Palomino, Aide Sandoval-Juarez, Marco Galarza-Pérez, José Alarcón-Guerrero, Rosa Guevara-Montero, Víctor Cárdenas-López

**Affiliations:** 1 Facultad de Ciencias Biológicas, Universidad Nacional San Cristóbal de Huamanga, Ayacucho 05001, Peru; mayramarisolmaldonadoaroni721@gmail.com (M.M.-A.); jose.alarcon@unsch.edu.pe (J.A.-G.); rosa.guevara@unsch.edu.pe (R.G.-M.); victor.cardenas@unsch.edu.pe (V.C.-L.); 2 Centro Nacional de Salud Pública, Instituto Nacional de Salud, Lima 150108, Peru; asandoval@ins.gob.pe (A.S.-J.); mgalarza@ins.gob.pe (M.G.-P.)

**Keywords:** *Leishmania*, Leishmaniasis, Real-Time PCR, Heat Shock Protein 20

## Abstract

Leishmaniasis is a complex neglected tropical disease that impacts public health, particularly in resource-limited populations where access to accurate and timely diagnosis is often limited. Current diagnostic methods, primarily relying on microscopy, suffer from low sensitivity and specificity, hindering effective case management and disease control. The objective of this study was to validate a novel real-time PCR assay targeting the conserved *Hsp20* gene for the detection of *Leishmania* spp. We evaluated the performance of the method using two distinct detection systems, such as SYBR Green and TaqMan probes, against a diverse panel of 225 clinical samples confirmed to have the disease. The real-time PCR targeting *Hsp20* using SYBR Green demonstrated a sensitivity of 88% (95% CI: 83.53–92.47) and 100% specificity. Meanwhile, the TaqMan probe demonstrated a lower sensitivity of 47% (95% CI: 29.53–64.92). The high sensitivity and robust performance of the real-time PCR using SYBR Green establish its potential as a valuable diagnostic tool, particularly useful in endemic regions where rapid and accurate diagnosis is critical for timely treatment and effective disease control.

## 1. Introduction

Leishmaniasis is a vector-borne disease that is prevalent worldwide, particularly in countries with tropical and subtropical climates. Annually, it is estimated that 30,000 new cases of visceral leishmaniasis and over 1 million new cases of cutaneous leishmaniasis occur. The clinical presentation of leishmaniasis depends on the species of *Leishmania* that infects and the immune status of the host. The disease can manifest in three main forms: visceral, cutaneous, and mucosal [1]. In the Americas, leishmaniasis is present in 18 countries, with more than 53,000 new cases recorded annually. The countries with the highest prevalence of the disease are Brazil, Colombia, and Peru [2].

Of the more than 20 species that cause the disease, about 13 are endemic to the Americas. Of these, only *Leishmania (Leishmania) infantum* is a viscerotropic species found in 13 countries in the region, including Argentina, Bolivia, Colombia, Costa Rica, El Salvador, Guatemala, Honduras, Mexico, Nicaragua, Paraguay, Uruguay, Venezuela, and Brazil, the latter accounting for more than 95% of the total number of cases. In contrast, Brazil, Colombia, and Peru account for over 70% of the cases of the American tegumentary leishmaniasis. The most frequently reported species are *Leishmania (Viannia) braziliensis*, *Leishmania (Viannia) guyanensis*, *Leishmania (Viannia) panamensis*, *Leishmania (Viannia) peruviana*, *Leishmania (Viannia) lainsoni,* and *Leishmania (Leishmania) mexicana* [3,4,5,6].

A definitive diagnosis of the disease is reached through the application of parasitological methods, including direct microscopic examination (DME) by smear or impression, *in vitro* culture, microculture, and histopathology. The sensitivity and specificity of these methods have been reported to vary between 60% and 100% [7]. Conversely, molecular methods based on the amplification of nucleic acids have facilitated the evaluation of a number of genetic markers, including *18S rDNA*, *kDNA*, *Hsp70*, *ITS1* and *ITS2*, and *AAP3*, among others [8,9], all of which are directed towards the detection of the genus *Leishmania*. However, only a limited number of studies have evaluated their suitability for a significant number of clinical samples [10,11,12,13].

Among the targets evaluated, the *heat shock protein 20* gene (*Hsp20*), identified in *Leishmania major*, *Leishmania infantum*, and *Leishmania braziliensis*, showed high conservation among *Leishmania* species but divergence from other organisms. This enabled the development of a conventional PCR assay targeting *Hsp20*, demonstrating superior analytical sensitivity and high diagnostic specificity across all *Leishmania* species [14,15,16]. Our objective was to develop and validate a real-time PCR assay targeting the *Hsp20* gene, given its advantages such as sensitivity, specificity, and throughput.

In the present study, real-time PCR targeting the *Hsp20* region was evaluated for the detection of *Leishmania* species using SYBR Green and TaqMan probes with DNA from six different *Leishmania* strains. The real-time PCR method was validated on tissue samples from patients with confirmed leishmaniasis, obtained from Giemsa-stained slides and lesion scrapings collected with lancets.

## 2. Materials and Methods

### 2.1. Reference Strains

The present retrospective observational study was conducted at the Laboratorio de Metaxénicas y Zoonosis Parasitaria of the Instituto Nacional de Salud (INS), Perú. The study focused on *Leishmania* strains belonging to the subgenus *Viannia* and *Leishmania* species (Table 1), which were cryopreserved in liquid nitrogen; the strains were thawed and cultured in a biphasic medium consisting of blood agar and Schneider’s liquid medium (Ref. 21720024, Gibco, Paisley, UK) with 20% heat-inactivated fetal bovine serum (HI-FBS) (Ref 10082-147, Gibco, Grand Island, NY, USA) and an antibiotic-antimycotic solution (Ref. 15240-062, Gibco, Carlsbad, CA, USA) (supplemented Schneider’s medium). Daily microscopic observation was employed during the reactivation process.

The medium containing *Leishmania* parasites was subjected to centrifugation at 8000 rpm for 5 min. The supernatant was then carefully removed, and the sediment was retained for genomic DNA extraction. The DNA samples from the strains were confirmed by *Hsp70* PCR-RFLP, using the primers *Hsp70*sen 5′-GACGGTGCCTGCCTGCCTACTACTTCAA-3′ and *Hsp70*ant 5′-CCGCCCATGCTCTCTGGTACATC-3′ [17,18]. The methodology previously described by Fraga et al. (2012) was employed, utilizing the restriction enzymes HaeIII and RsaI (Cat. ER0151 and ER1122, Thermo Scientific, Vilnius, Lithuania).

### 2.2. Sample Collection

The tissue samples included in this study were obtained between the years 2011 and 2019, as part of the process for the confirmatory diagnosis of the disease in patients who were referred to the National Institute of Health, or in the context of the quality control process for the direct microscopic examination conducted by the National Reference Laboratory for Metaxenic and Parasitic Zoonoses and implemented by regional reference laboratories.

The sample size was determined using the free software EPIDAT 4.2 with an expected sensitivity and specificity greater than 95% [16]; we used a precision of 5%, a healthy-to-sick ratio of 1, and a 95% confidence interval (CI), employing the following formulas:

$$n=\frac{Z^{2}\;(S)(1-S)}{L^{2}}\;\;\;\;\;and\;\;\;\;\;n=\frac{Z^{2}\;(E)(1-E)}{L^{2}}$$

where *n* represents the sample size, *Z* denotes the confidence level, *S* corresponds to the expected sensitivity, *E* corresponds to the expected specificity, and *L* represents the acceptable margin of error.

Samples were selected for convenience (see Supplementary Material Tables S1 and S2) according to the inclusion and exclusion criteria for the details of the groups:

Group I included Giemsa-stained slides sent by local laboratories in endemic areas to the Laboratorio de Referencia Nacional de Metaxénicas y Zoonosis Parasitaria for quality control in direct microscopic examination. Only slides that showed positive results by visualizing at least one amastigote form in one hundred fields using a 100x oil-immersion objective was included. Additionally, Giemsa-stained slides that showed negative results, a low presence of tissue, poor conservation quality, or evidence of contamination were excluded from the group.

Group II included tissue samples obtained from patients by scraping with sterile lancets, which were used to prepare Giemsa-stained slides for direct microscopic examination or to inoculate a biphasic medium for in vitro culture. These samples showed positive results, either by visualizing at least one amastigote form in one hundred fields using the 100x oil-immersion objective, by visualizing promastigote forms in liquid medium with an inverted microscope using a 40x objective, or by both methods. The tissue obtained from the lancets was preserved in sterile polypropylene conical tubes immersed in 96% alcohol.

Group III included tissue samples from lancets of patients who showed negative results by assessing all fields of the Giemsa-stained slides in direct microscopic examination with a 100x oil-immersion objective, or who did not exhibit promastigote forms in liquid medium when evaluated using an inverted microscope with a 40x objective. Additionally, samples from patients who showed a positive result when assessed in the Montenegro skin test were excluded.

To ensure a rigorous analysis process and minimize bias, samples failing to meet the inclusion criteria for any group were excluded. Furthermore, the remaining samples underwent initial assessment for human *RNase P* detection using real-time PCR before *Hsp20* assessment. Samples yielding neither human *RNase P* nor *Hsp20* amplification were excluded from the final analysis.

### 2.3. DNA Extraction and Purification

Genomic DNA was extracted from the samples using the Purelink Genomic DNA Mini Kit (Ref. K1820-02, Invitrogen, Life Technologies, Carlsbad, CA, USA). Reference strains were processed in accordance with the kit instructions and were eluted in a final volume of 100 µL.

Prior to genomic DNA extraction, the slides were immersed in methanol to eliminate any residual dye. The tissue samples from each slide were removed individually with the aid of a No. 15 scalpel blade. The biological material was transferred to conical polypropylene tubes and subjected to centrifugation at 8000 rpm for 10 min. Subsequently, the supernatant was then removed and 180 µL of the PureLink^TM^ Genomic Digestion Buffer was added.

To extract DNA from tissue obtained by lancing, the tubes containing the samples were subjected to centrifugation at 8000 rpm for 10 min. Subsequently, the supernatant was removed and 180 µL of the digestion buffer was added.

The tubes containing the samples, obtained from either Giemsa-stained slide or lancets, were homogenized and incubated at 55 °C for two hours. Subsequently, the samples were processed in a manner like that used for the extraction of genomic DNA from *Leishmania* strains. The DNA was quantified using an Eon5 spectrophotometer (BioTek Instruments, Winooski, VT, USA), and then aliquoted to a volume of 50 µL for storage at −20 °C until required.

### 2.4. Primer Design and Performance Evaluation of Real-Time PCR

The real-time PCR targeting the region of the gene encoding *Hsp20* was developed using the primers initially described by Montalvo A., et al. (2020): *Hsp20*S F: 5′-GCCRGARGTGARRAAGGAGGAGGAC-3′ and *Hsp20*S R: 5′-GYAGCTGGYKYTCGTCCTGC-3′. Likewise, a specific FAM/TAMRA-linked probe sequence was designed, using the program Geneious Prime 2022.1 based on the sequences of *Leishmania (Leishmania) aethiopica* JX630112, *Leishmania (Leishmania) tropica* JX630114, *Leishmania (Leishmania) major* JX630117, *Leishmania (Leishmania) donovani* JX630120, *Leishmania (Leishmania) infantum* JX630122, *Leishmania (Leishmania) chagasi* JX630125, *Leishmania (Leishmania) mexicana* JX630126, *Leishmania (Leishmania) amazonensis* JX630128, *Leishmania (Leishmania) garnhami* JX630129, *Leishmania (Viannia) braziliensis* JX630135, *Leishmania (Viannia) peruviana* JX630139, *Leishmania (Viannia) guyanensis* JX63011242, *Leishmania (Viannia) panamensis* JX63011243, and *Leishmania (Viannia) lainsoni* JX630148 from the GenBank database. Real-time PCR targeting *Hsp20* was evaluated on MicPCR (Biomolecular Systems, Upper Coomera, Australia) and Rotor-Gene Q (Qiagen, Hilden, Germany) thermal cyclers in a reaction volume of 10 µL, as well as during the standardization process.

The performance of real-time PCR for the detection of a specific region of *Hsp20* of *Leishmania* was evaluated using an intercalating fluorophore with an affinity for DNA (SYBR Green), as well as Taqman-labeled probes.

To detect *Leishmania* with intercalating fluorophores, a 1x solution of Luminaris Color HiGreen qPCR Master Mix 2x solution (Cat. K0392, Thermofisher, Vilnius, Lithuania) was employed, along with primer *Hsp20*S F and *Hsp20*S R at 0. 3 µM each and 3 µL of genomic DNA. The total volume was completed with molecular biology-grade water. The amplification conditions included an initial denaturation step at 95 °C for five minutes for polymerase activation. Subsequently, two stages were conducted. The initial stage comprised five cycles without data acquisition: denaturation at 95 °C for 15 s, followed by a hybridization/extension at 62 °C for 30 s. The second stage comprised 45 cycles with data acquisition, which consisted of denaturation at 95 °C for 15 s and hybridization/extension at 62 °C for 30 s.

The detection of *Leishmania* using Taqman-labeled probes was achieved using 1x Platinum™ Quantitative PCR SuperMix-UDG 2x (Invitrogen, Life Technologies, Carlsbad, CA, USA), primers *Hsp20*S F and *Hsp20*S R at 0.5 µM, and probe *Hsp20*SS at 0.3 µM. The amplification conditions were similar to those used with the intercalating fluorophore, with the exception that the hybridization/extension step was developed at 57 °C. The final concentration of the magnesium chloride in the reaction tubes was 3.5 mM, the concentration of the primer and probe was 0.3 µM, and the volume of DNA was 3 µL. The remaining components were molecular biology-grade water.

### 2.5. Real-Time PCR Efficient, Analytical Sensitivity, Inclusivity, and Exclusivity

To determine the efficiency of the real-time PCR, as well as the analytical sensitivity (limit of detection, LoD) of the method, genomic parasite DNA was prepared at concentrations equivalent to 1 × 10^4^ up to 1 × 10^−1^, parasite/mL by serial dilution from different *Leishmania* species [7,19].

The inclusivity was determined by the detection of *Leishmania* species described in Table 1, while exclusivity was evaluated using genomic DNA samples from related pathogens, including *Crithidia* spp., *Trypanosoma cruzi*, *Plasmodium vivax*, and *Plasmodium falciparum*. All parameters were assessed in triplicate.

### 2.6. Validation Process

The validation of the real-time PCR method for the detection of *Leishmania* included the parameters of diagnostic sensitivity and specificity, predictive values, and precision of the method. All parameters were determined in accordance with the current Peruvian standards [20].

To guarantee the adequate DNA extraction process, the samples were previously processed for human *RNase P* region detection through real-time PCR. The TaqMan^®^ *RNase P* Detection Reagents Kit (Cat. 4316831, ThermoFisher Scientific, Vilnius, Lithuania) was used. The amplification was developed with denaturation at 95 °C for 2 min, followed by 40 cycles of denaturation at 95 °C for 3 s and hybridization/extension at 60 °C for 30 s. The data acquisition was performed using the green channel. A cutoff cycle threshold (Ct) value of ≤35 was established to minimize possible nonspecific amplifications and decrease the occurrence of false positives.

Samples were processed using real-time PCR, and those that amplified or exhibited a Ct value ≤ 35 were considered positive. In this context, samples that amplified solely for the *Hsp20* region or for both *Hsp20* and *RNase P* were classified as positive. Samples that amplified human *RNase P* but did not achieve a Ct value of 35 or lower for the *Hsp20* target were deemed negative. Conversely, samples that did not amplify any targets for either *Hsp20* or human *RNase P* were excluded from subsequent analysis.

The diagnostic sensitivity and specificity, along with the predictive values and likelihood ratios, were determined through the evaluation of groups I, II, and III. The repeatability of the method was assessed by processing a positive and a negative control sample on five separate occasions over a five-day period, under the same conditions concerning the analyst, instrument, and environment.

Similarly, intermediate precision was determined from the results by two analysts who employed the same methodology utilized for repeatability.

### 2.7. Analysis

Efficiency of real-time PCR was determined using the following formula [19]:

$$Efficiency\;\left(E\right)=10^{-\left(\frac{1}{slope}\right)}-1$$

In this context, the slope reflects the steepness of the standard curve, as determined by plotting the Ct values against the logarithm of the starting quantity of target DNA [19].

The sensitivity, specificity, positive predictive value (PPV), negative predictive value (NPV), likelihood ratio (LR), and area under the curve (AUC) were determined using the parasitological tests as the reference standards. Statistical analysis was conducted using Fisher’s exact test in SPSS v.26.0 software, with a confidence interval of 95%.

### 2.8. Ethics

The present study was developed from samples that were obtained between 2011 and 2019 within the framework of the specialized diagnosis of the disease in patients and the Quality Control Program of the Direct Microscopic Examination of the National Institute of Health. Furthermore, the study was approved by the Ethics in Research Committee of Instituto Nacional de Salud—Peru, by Memorandum No. 028-2022-CIEI-INS.

## 3. Results

Real-time PCR targeting the *Hsp20* region was optimized for *Leishmania* detection. A slope ranging from −3.40 to −3.89 was observed when SYBR Green was used, with lower values for *Leishmania (Leishmania) mexicana* and higher values for *Leishmania (Leishmania) amazonensis*. Additionally, R^2^ values were determined between 0.94 and 0.99, and an efficiency of the real-time PCR greater than 90%.

**Figure 1:**
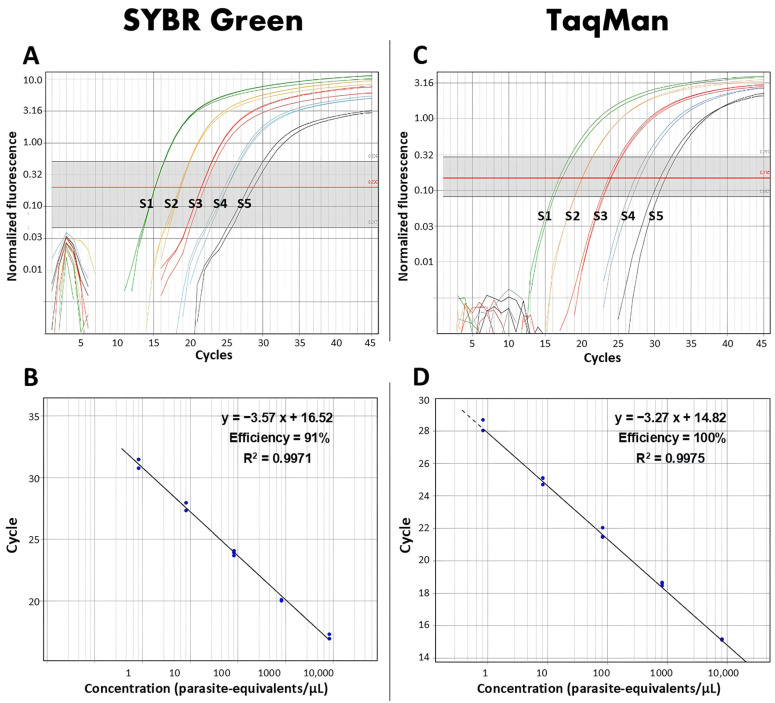
Evaluation of the efficiencies of real-time PCR targeting *Hsp20* based on standard curves of *Leishmania (Viannia) guyanensis* generated using SYBR Green (**A**) and TaqMan (**C**) detection systems. Amplification efficiencies were determined for both methods (**B**,**D**). S1 to S5 represent the serial dilutions of 10^4^, 10^3^, 10^2^, 10, and 1 parasite/µL used to construct the standard curves.

In contrast, the TaqMan probe exhibited a slope ranging from −3.27 to −4.096, with lower values for *Leishmania (Viannia) guyanensis* and higher values for *Leishmania (Viannia) lainsoni*, respectively, with R^2^ values of 0.99, and an efficiency of 100%, as shown in Figure 1 and Table 2.

Similarly, the LoD was found to be 1 parasite/µL in both systems. However, *Leishmania (Viannia) peruviana* was not amplified using the TaqMan probe, indicating a limitation in the detection range of this specific probe compared to using SYBR Green. 

Regarding the inclusivity of the method, all species of *Leishmania* described in Table 1 were detected using SYBR Green, whereas with the TaqMan probe, *Leishmania (Viannia) peruviana* did not show amplification. With respect to exclusivity, both detection systems demonstrated specificity for *Leishmania*, while neither detection system exhibited amplification when sourced from DNA from *Crithidia* spp., *Plasmodium vivax*, *Plasmodium falciparum*, and *Trypanosoma cruzi*.

In the validation process, a total of 323 samples were included in this study. Specifically, 128 samples from Giemsa-stained slides were categorized as Group I, while 129 samples obtained from lancets were classified as Group II. Additionally, 66 tissue samples from lancets identified as negative for *Leishmania* were categorized as Group III.

During the analysis process, 32 samples were excluded based on specific criteria to ensure reliability and accuracy. These excluded samples exhibited a Ct value for the *RNase P* that exceeded a Ct value of 35. Of these excluded samples, 15 were from Group I, and the remaining 17 were from Group II.

The results are presented in Table 3. The real-time polymerase chain reaction (PCR) targeting the *Hsp20* region with SYBR Green was performed on a total of 225 samples that tested positive in parasitological assays. Of these, 198 were accurately identified, while 27 yielded false-negative results; of these, 17 were from Giemsa-stained slides and 10 from lancet samples. The overall sensitivity of the method was 88.00% (95% CI: 83.53–92.47), with sensitivities of 84.96% (95% CI: 77.9–91.9) and 91.20% (95% CI: 84.3–95.7) for Giemsa-stained slides and lancets, respectively. Importantly, no false positives were observed, leading to a specificity of 100%.

Conversely, given the limitations of *Leishmania (Viannia) peruviana* and *Leishmania (Viannia) lainsoni* detected by the TaqMan probe, this analysis was conducted on 36 samples that were randomly selected from Group II. Seventeen samples were identified as positive, while nineteen were classified as false negatives, resulting in a sensitivity of 47.22% (95% CI: 29.53–64.92). Notably, the TaqMan probe exhibited no false positives, as shown in Table 4.

**Figure 2:**
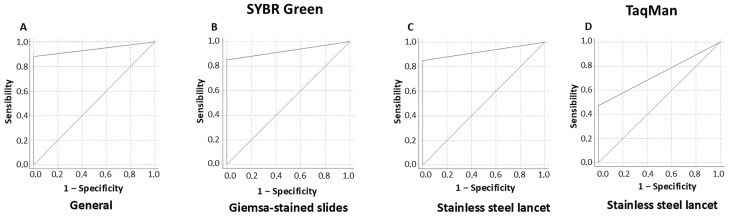
ROC curve representing real-time PCR targeting *Hsp20* with SYBR Green, considering both Giemsa-stained slides and stainless steel lancets (**A**), as well as independently (**B**,**C**), alongside the use of a TaqMan probe (**D**).

Finally, the AUC for real-time PCR targeting the *Hsp20* gene region using SYBR Green considering positive samples from both Giemsa-stained slides and lancets was 0.94 (95% CI: 0.91–0.97). Independently, samples from Giemsa-stained slides showed an AUC of 0.92 (95% CI: 0.88–0.97), while samples obtained from lancets demonstrated an AUC of 0.96 (95% CI: 0.93–0.98). In the case of the TaqMan probe, the method assessed using samples from lancets reached an AUC of 0.74 (95% CI: 0.62–0.85), as shown in Figure 2.

## 4. Discussion

To date, direct microscopic examination remains the most widely used method for diagnostic leishmaniasis, which involves the microscopic visualization of parasites from tissue samples that are fixed in glass slides and stained with Giemsa, allowing for time-efficient results. However, despite this advantage, this method presents limitations associated with sensitivity, which is closely related to the quality of the sample. Poor sample collection can lead to false-negative results.

Moreover, the performance of direct microscopic examination could be influenced by factors such as the stage of the disease, the clinical presentation, parasite loads, and other variables that significantly affect diagnostic outcomes. It is inherently limited by the subjective nature of the examination process. The specificity of the test depends mainly on the experience and skill of the microscopist [21]. Those with limited experience may confuse the parasitic form of *Leishmania* with other pathogens, such as *Histoplasma* or *Sporothrix schenckii*, leading to misdiagnosis and inappropriate management of the condition. On the other hand, patients with chronic cutaneous or mucosal lesions represent a greater diagnostic challenge because of their reduced parasitic load, which may result in false negatives, delayed treatment, and adverse patient outcomes [22,23].

Integrating molecular diagnostic techniques, such as PCR, can significantly improve sensitivity and specificity, even in samples with reduced parasitic load, thereby addressing one of the key limitations of microscopy. This approach can optimize patient management strategies, particularly in endemic regions. In this context, real-time PCR is a tool with increased sensitivity and specificity compared to conventional diagnostic methods, capable of detecting even low concentrations of parasitic DNA, down to a single copy, as demonstrated in this study with the *Hsp20* gene. This capability is especially important for the early and accurate diagnosis of patients with chronic skin lesions or those with mucosal lesions. As a result, the likelihood of false-negative results is significantly reduced. In this context, molecular methods can facilitate the diagnosis of the disease, such as real-time PCR targeting the *18S rDNA*, *kDNA*, *ITS1*, and *Hsp70* regions, among others, genetic markers for the detection of *Leishmania* [10,12,24,25].

The *Hsp20* gene, a conserved marker in *Leishmania*, was initially identified in *Leishmania amazonensis* in 2009 [14]. Previous reports have demonstrated that the target exhibits a high level of sensitivity and specificity in the detection of *Leishmania* when employing conventional PCR [15,16].

In this study, we utilized real-time PCR targeting *Hsp20* for the detection of *Leishmania*. We evaluated the efficiencies of the SYBR Green and TaqMan detection systems in terms of their ability to amplify the target DNA during each cycle [19], thereby ensuring accurate and consistent detection of *Leishmania* in the analyzed samples. Our findings revealed that the molecular method exhibits amplification efficiencies of 91% and 100%, respectively, while the LoD was of one parasite per microliter for both systems.

The estimated diploid genome size of *Leishmania* is 83.15 femtograms (fg). Previous reports have demonstrated that the real-time PCR that targets the SSU-rRNA region allows for the detection of up to 0.036 picograms (pg) of *Leishmania* DNA [24], equivalent to 36 fg. When compared with the total DNA concentration of *Leishmania*, this represents an ability to identify DNA quantities equivalent to less than one parasite. Meanwhile, the *18S rRNA* target allows for the detection of approximately 8.32 × 10^−3^ fg, predominantly in *Leishmania (Viannia) guyanensis* and *Leishmania (Viannia) panamensis*, which is equivalent to the total genomic DNA of a single parasite diluted to a ratio of 1:1000. In the case of *Leishmania (Viannia) braziliensis* and *Leishmania (Leishmania) amazonensis*, the assay exhibited an LoD of up to one parasite [10].

Similar to the 18S rRNA, the Hsp70 gene region utilized in real-time PCR enabled the detection of up to one parasite in Leishmania (Viannia) guyanensis, Leishmania (Viannia) braziliensis, and Leishmania (Leishmania) amazonensis. Leishmania (Viannia) panamensis could be detected at a concentration equivalent to 1000 parasites [10]. In another study, both 18S rRNA and kDNA demonstrated the ability to detect a thousandths part of a parasite, while Hsp70 and ITS were able to detect a concentration equivalent to a hundredth of Leishmania [9].

In relation to inclusivity, the assessed *Hsp20* region in real-time PCR using SYBR Green exhibited the ability to detect different *Leishmania* species, which is concordant with targets such as *Hsp70*, *kDNA*, *ITS*, and *18S rRNA*. Conversely, the use of a TaqMan probe did not show the ability to detect *Leishmania (Viannia) peruviana*; additionally, *Leishmania (Viannia) lainsoni* yielded a slope of −4.0. This characteristic was possibly related to specific variations in the complementary DNA sequences of the TaqMan probe.

The *Hsp20* region in both detection systems (SYBR Green and TaqMan probe) were shown to be specific to *Leishmania*, as demonstrated in studies developed by Montalvo A (2020), a characteristic similar to that of *Hsp70* and *ITS*. Meanwhile, the *18S rRNA* and *kDNA,* which demonstrated an ability to detect quantities lower than one parasite, exhibited cross-reactivity, particularly with *Trypanosoma cruzi* [9,10].

During the validation process, we found that the detection of *Leishmania* spp. by real-time PCR targeting the *Hsp20* region exhibited 100% specificity (95% CI: 99.2–100), with no significant differences compared to the gold standard, consistent with previous reports [26]. Conversely, the sensitivity of the molecular method showed a significant difference when the SYBR Green system and the TaqMan probe were compared.

Sensitivity values for *Hsp70* and *18S rRNA* were reported at 83.6% and 90.9%, respectively, accompanied by a specificity of 61% for Hsp70 and 18% for *18S rRNA* in real-time PCR using the TaqMan probe [10]. In the case of *kDNA*, sensitivity values ranged from 73% to 88% using SYBR Green and from 54% to 79% with the TaqMan probe, both demonstrating specificity values of 100% [27,28]. Our real-time PCR targeting the *Hsp20* gene region using SYBR Green reached a sensitivity value of 88% (95% CI 83.53–92.47) with a specificity of 100% (95% CI 94.2–100).

Using SYBR Green, a total of 27 samples were identified as false negatives; 17 DNA samples from Giemsa-stained slides exhibited discordant results compared to the reference parasitological results. Additionally, ten samples from tissue obtained from lancets were also false negatives. This mismatch did not correlate with the parasite load and is probably associated with the preservation of the DNA samples or a probable genetic variability of *Leishmania*, which does not allow adequate hybridization between the oligonucleotide sequence of the labeled TaqMan probe and the specific sequence of *Leishmania.*

In contrast, the use of the TaqMan probe reported sensitivity and specificity values of 47% (95% CI 29.52–64.92) and 100% (95% CI 99.24–100.00), respectively. The limited clinical sensitivity could be attributed to variations in the sequences of certain *Leishmania* species, mainly *Leishmania (Viannia) peruviana* and *Leishmania (Viannia) lainsoni*. Using this detection system, from 36 samples randomly selected from Group II, 19 samples identified as false negatives. This mismatch was probably associated with the genetic variability of the parasite that limits the capacity of the TaqMan probe to detect all species of *Leishmania*. These difficulties underscore the need for a new design of the TaqMan probe to improve the performance of the method.

These sensitivity and specificity values of the real-time PCR-*Hsp20* method for the detection of *Leishmania* found using SYBR Green were comparable to those previously reported by Montalvo A. et al. (2020), despite differences in the number of samples used in each study. These results demonstrated the robustness of the real-time PCR-*Hsp20* method. The analysis showed that the sensitivity observed in the samples obtained from lancets was slightly higher than in the DNA samples from Giemsa-stained slides; however, this difference was not statistically significant (*p*-value > 0.05), suggesting that both sample types allow for the effective detection of *Leishmania*. This slight variation could be related to the conservation of tissue samples and DNA in Giemsa-stained slides compared to samples from lancets that were preserved in alcohol, which allows for better conservation of tissue samples and DNA.

## 5. Conclusions

In conclusion, the real-time PCR method targeting the *Hsp20* gene using SYBR Green for the detection of *Leishmania* spp. has demonstrated high sensitivity and specificity, achieving values of 88% and 100%, respectively, in DNA samples extracted from DME slides and lancets. This method can detect low concentrations of *Leishmania* down to a single cell, which underscores its potential for early and accurate diagnosis, which is particularly important for patients presenting with chronic cutaneous lesions or mucosal involvement, where the parasite load may be minimal and traditional diagnostic methods may fall short.

Furthermore, the application of this diagnostic method could facilitate access to timely treatment, significantly improving patient outcomes, as well as initiating appropriate strategies for controlling the disease in conditions where progression may lead to severe complications.

However, it is essential to continue research focused on identifying specific sequences associated with the TaqMan probe that enhance the method and enable the development of a duplex real-time PCR assay, allowing for the simultaneous detection of both the *Hsp20* region and an endogenous control. This dual-target approach will not only improve diagnostic precision but also assess the quality of the samples being tested, contributing to the accuracy of the method.

Additionally, further investigations should aim to validate the real-time PCR-*Hsp20* method in larger and more diverse populations to ensure its applicability across various demographic groups and clinical presentations. Expanding the scope of the study will help ascertain the method’s robustness and adaptability to different geographical settings.

## Figures and Tables

**Table 1:** Strains of *Leishmania* spp. and other pathogens used in the evaluation of real-time PCR targeting *Hsp20*.

Name	Code
*Leishmania (Viannia) braziliensis*	MHOM/BR/75/M2904
*Leishmania (Viannia) peruviana*	MHOM/PE/90/LCA08
*Leishmania (Viannia) guyanensis*	MHOM/BR/70/M1176
*Leishmania (Viannia) lainsoni*	MHOM/PE/88/BAB1730
*Leishmania (Leishmania) amazonensis*	MHOM/BR/73/M2269
*Leishmania (Leishmania) mexicana*	MNYC/BZ/62/M379

**Table 2:** Parameters of real-time PCR targeting *Hsp20* using intercalating fluorophore and probe labeled against reference strains.

No.	Specie	SYBR Green (Threshold 0.15)			Taqman (Threshold 0.2)		
		Slope (M)	Y-Intercept	R^2^	Slope (M)	Y-Intercept	R^2^
1	*L. (V.) braziliensis*	−3.512	13.684	0.98068	−3.408	27.51	0.9999
2	*L. (V.) peruviana*	−3.512	13.684	0.98068	Not detected		
3	*L. (V.) guyanensis*	−3.57	16.520	0.9971	−3.27	14.82	0.9975
4	*L. (V.) panamensis*	ND			−3.314	27.2	0.9999
5	*L. (V.) lainsoni*	−3.564	18.074	0.99112	−4.096	32.72	0.9914
6	*L. (L.) mexicana*	−3.406	21.175	0.94880	ND		
7	*L. (L.) amazonensis*	−3.890	22.012	0.98663	−3.438	27.68	0.9941
8	*Trypanosoma cruzi*	Not detected			Not detected		
9	*Plasmodium vivax*	Not detected			Not detected		
10	*Plasmodium falciparum*	Not detected			Not detected		
11	*Crithidia* spp.	Not detected			Not detected		

ND: Not determined.

**Table 3:** Results of real-time PCR targeting *Hsp20* using an intercalating fluorophore.

	SYBR Green		
	General 95% CI *n* = 291	Giemsa-Stained Slides 95% CI *n* = 113	Lancets 95% CI *n* = 178
True positive	198/225	96/113	102/112
False positive	0	0	0
True negative	66/66	-	66/66
False negative	27	17	10
Sensitivity	88.00 (83.53–92.47)	84.96 (77.9–91.9)	91.2 (84.3–95.7)
Specificity	100 (99.24–100)	100 (94.2–100)	100 (94.6–100)
AUC	0.94 (0.91–0.97)	0.92 (0.88–0.97)	0.96 (0.93–0.98)
PPV	100 (99.75–100)	100 (99.48–100)	100 (96.5–100)
NPV	70.97 (61.20–80.73)	70.52 (70.23–88.80)	86.8 (77.1–93.5)
LR (+)	-	-	-
LR (−)	0.12 (0.08–0.17)	0.15 (0.10–0.23)	0.09 (0.05–0.17)

AUC: area under curve; PPV: positive predictive value; NPV: negative predictive value; LR: likelihood ratio.

**Table 4:** Results of real-time PCR targeting *Hsp20* using a labeled probe.

	TaqMan
	Lancets (95%CI) *n* = 102
True positive	17/36
False positive	0
True negative	66/66
False negative	19
Sensibility	47.22 (29.53–64.92)
Specificity	100 (99.24–100.00)
AUC	0.74 (0.62–0.85)
PPV	100 (97.06–100.00)
NPV	77.65 (68.20–87.09)
LR (+)	-
LR (−)	0.53 (0.39–0.72)

AUC: area under curve; PPV: predictive positive value; NPV: negative predictive value; LR: likelihood ratio.

## Data Availability

Study data are available from the corresponding author upon request.

## Supplementary Materials

The following supporting information can be downloaded at: https://www.mdpi.com/article/10.3390/tropicalmed10050121/s1, Table S1: Characteristics of clinical samples of confirmed patients included in the study evaluated using intercalating fluorophore; Table S2: Characteristics of the clinical samples of confirmed patients included in the study evaluated using a marked probe.

## References

[1] World Health Organization (2024). Leishmaniasis. https://www.who.int/health-topics/leishmaniasis.

[2] WHO-PAHO (2021). Leishmaniasis: Informe Epidemiológico de las Américas No. 10, Diciembre 2021. Inf. Leishmaniasis.

[3] WHO-PAHO (2020). Leishmaniasis: Informe Epidemiológico de las Américas. No. 9, Diciembre del 2020. Inf. Leishmaniasis.

[4] Van der Auwera G., Dujardin J.-C. (2015). Species typing in dermal leishmaniasis. Clin. Microbiol. Rev..

[5] Sheikh S.S., Amir A.A., Amir B.A., Amir A.A., Bastidas G., Kamboh A.A. (2020). Leishmaniasis. Parasitology and Microbiology Research.

[6] Ministério da Saúde (2017). Manual de Vigilância da Leishmaniose Tegumentar.

[7] León C.M., Muñoz M., Tabares J.H., Hernandez C., Florez C., Ayala M.S., Ramírez J.D. (2018). Analytical performance of a loop-mediated isothermal amplification assay for leishmania DNA detection in sandflies and direct smears of patients with cutaneous leishmaniasis. Am. J. Trop. Med. Hyg..

[8] Akhoundi M., Downing T., Votýpka J., Kuhls K., Lukeš J., Cannet A., Ravel C., Marty P., Delaunay P., Kasbari M. (2017). Leishmania infections: Molecular targets and diagnosis. Mol. Asp. Med..

[9] León C.M., Muñoz M., Hernández C., Ayala M.S., Flórez C., Teherán A., Cubides J.R., Ramírez J.D. (2017). Analytical performance of Four Polymerase Chain Reaction (PCR) and real time PCR (qPCR) assays for the detection of six Leishmania species DNA in Colombia. Front. Microbiol..

[10] Filgueira C.P.B., Moreira O.C., Cantanhêde L.M., de Farias H.M.T., Porrozzi R., Britto C., Boité M.C., Cupolillo E. (2020). Comparison and clinical validation of qPCR assays targeting Leishmania 18S rDNA and HSP70 genes in patients with American Tegumentary Leishmaniasis. PLoS Negl. Trop. Dis..

[11] Montalvo Alvarez A.M., Fraga J., Tirado D., Blandón G., Alba A., Van der Auwera G., Vélez I.D., Muskus C. (2017). Detection and identification of *Leishmania* spp.: Application of two hsp70-based PCR-RFLP protocols to clinical samples from the New World. Parasitol. Res..

[12] Wu Y., Tian X., Song N., Huang M., Wu Z., Li S., Waterfield N.R., Zhan B., Wang L., Yang G. (2020). Application of Quantitative PCR in the Diagnosis and Evaluating Treatment Efficacy of Leishmaniasis. Front. Cell. Infect. Microbiol..

[13] Tellevik M.G., Muller K.E., Løkken K.R., Nerland A.H. (2014). Detection of a broad range of Leishmania species and determination of parasite load of infected mouse by real-time PCR targeting the arginine permease gene AAP3. Acta Trop..

[14] Montalvo-Álvarez A.M., Folgueira C., Carrión J., Monzote-Fidalgo L., Cañavate C., Requena J.M. (2008). The *Leishmania* HSP20 is antigenic during natural infections, but, as DNA vaccine, it does not protect BALB/c mice against experimental *L. amazonensis* infection. J. Biomed. Biotechnol..

[15] Montalvo A.M., Fraga J., Rodríguez O., Blanco O., Llanos-Cuentas A., García A.L., Valencia B.M., Muskus C., Van der Auwera G., Requena J.M. (2014). Detección de *Leishmania* spp. en base al gen que Codifica la Proteína HSP20. Rev. Peru Med. Exp. Salud Publica.

[16] Montalvo A.M., Alba A., Fraga J., Marzoa A., Torres C., Muskus C. (2020). Improving the sensitivity of an Hsp20-based PCR for genus detection of Leishmania parasites in cutaneous clinical samples: A proof of concept. Parasitol. Res..

[17] Apaza-Castillo Y.G., Aguilar-Ancori E.G., Quispe-Florez M.M., Ramirez-Soto M.C., Pacheco-Venero R.L. (2020). PCR performance for the diagnosis of cutaneous leishmaniasis caused by Leishmania viannia complex using biopsy samples, compared with exudate samples from skin lesions on filter paper. Trans. R. Soc. Trop. Med. Hyg..

[18] Fraga J., Veland N., Montalvo Alvarez A.M., Praet N., Boggild A.K., Valencia B.M., Arévalo J., Llanos-Cuentas A., Dujardin J.-C., Van der Auwera G. (2012). Accurate and rapid species typing from cutaneous and mucocutaneous leishmaniasis lesions of the New World. Diagn. Microbiol. Infect. Dis..

[19] Svec D., Tichopad A., Novosadova V., Pfaffl M.W., Kubista M. (2015). How good is a PCR efficiency estimate: Recommendations for precise and robust qPCR efficiency assessments. Biomol. Detect. Quantif..

[20] Instituto Nacional de Calidad (2020). Directive for the Validation and Verification of Qualitative Analysis Procedures in the Clinical Laboratories.

[21] Rojas-Palomino N., Sandoval-Juarez A., Solis-Sánchez G., Minaya-Gómez G. (2024). Rendimiento diagnóstico de los antígenos de *Leishmania braziliensis* y *Leishmania peruviana* en el método de inmunoblot para la detección de la leishmaniasis tegumentaria americana. Rev. Peru. Med. Exp. Salud Publica.

[22] de Jesus Oliveira Gonçalves C.A., Carneiro J.T., de Souza Cruz E.L., de Sousa Neves Filho F., Rivadeneira Cárdenas R.C., Guimarães D.M. (2020). Parasitological association between human leishmaniosis mucosa and paracoccidioidomycosis. Case report. Int. J. Surg. Case Rep..

[23] Al-Jawabreh A., Dumaidi K., Ereqat S., Nasereddin A., Azmi K., Al-Jawabreh H., Al-Laham N., Abdeen Z. (2018). A comparison of the efficiency of three sampling methods for use in the molecular and conventional diagnosis of cutaneous leishmaniasis. Acta Trop..

[24] Gomes L.I., Gonzaga F.M., de Morais-Teixeira E., de Souza-Lima B.S., Freire V.V., Rabello A. (2012). Validation of quantitative real-time PCR for the in vitro assessment of antileishmanial drug activity. Exp. Parasitol..

[25] Suárez M., Valencia B.M., Jara M., Alba M., Boggild A.K., Dujardin J.-C., Llanos-Cuentas A., Arévalo J., Adaui V. (2015). Quantification of Leishmania (Viannia) Kinetoplast DNA in Ulcers of Cutaneous Leishmaniasis Reveals Inter-site and Inter-sampling Variability in Parasite Load. PLoS Negl. Trop. Dis..

[26] Merdekios B., Pareyn M., Tadesse D., Eligo N., Kassa M., Jacobs B.K.M., Leirs H., Van Geertruyden J.P., van Griensven J., Caljon G. (2021). Evaluation of conventional and four real-time pcr methods for the detection of leishmania on field-collected samples in Ethiopia. PLoS Negl. Trop. Dis..

[27] Gomes C.M., Cesetti M.V., De Paula N.A., Vernal S., Gupta G., Sampaio R.N.R., Roselino A.M. (2017). Field validation of SYBR Green- and TaqMan-based real-time PCR using biopsy and swab samples to Diagnose American Tegumentary Leishmaniasis in an Area Where Leishmania (Viannia) braziliensis is endemic. J. Clin. Microbiol..

[28] Moreira O.C., Yadon Z.E., Cupolillo E. (2018). The applicability of real-time PCR in the diagnostic of cutaneous leishmaniasis and parasite quantification for clinical management: Current status and perspectives. Acta Trop..

