# Supplementary material for: Validation of a Real-Time PCR for the Diagnosis of Leishmania Species Using the Hsp20 Gene
Source: Trop Med Infect Dis. 2025 May 1;10(5):121. doi: 10.3390/tropicalmed10050121 (PMC12115578; doi:10.3390/tropicalmed10050121)
Supplement: Supplementary file 1 [file tropicalmed-10-00121-s001.zip › Supplementary material Table S1.pdf]

Table S1. Characteristics of clinical samples of confirmed patients included in the study evaluated using intercalating fluorophore.

N°	Code	Sample	Gold Standard result	DME Result	Culture result	Ct RNAseP	RNAseP result	Ct_Hsp20	Hsp20 result
**1**	ANCH-02	Giemsa-staining slide	Positive	Positive	-	33.19	Positive	0.00	Negative
**2**	CN-03 (2)	Giemsa-staining slide	Positive	Positive	-	31.62	Positive	0.00	Negative
**3**	CN-18	Giemsa-staining slide	Positive	Positive	-	32.11	Positive	0.00	Negative
**4**	HRA-39	Giemsa-staining slide	Positive	Positive	-	30.62	Positive	0.00	Negative
**5**	HRA-42	Giemsa-staining slide	Positive	Positive	-	29.47	Positive	0.00	Negative
**6**	LL-18	Giemsa-staining slide	Positive	Positive	-	32.77	Positive	40.45	Negative
**7**	LL-60	Giemsa-staining slide	Positive	Positive	-	33.22	Positive	0.00	Negative
**8**	LLOA-05	Giemsa-staining slide	Positive	Positive	-	32.08	Positive	0.00	Negative
**9**	SM-05	Giemsa-staining slide	Positive	Positive	-	32.40	Positive	0.00	Negative
**10**	SM-06	Giemsa-staining slide	Positive	Positive	-	31.53	Positive	43.86	Negative
**11**	SR-19	Giemsa-staining slide	Positive	Positive	-	31.95	Positive	40.91	Negative
**12**	LL-06 (2)	Giemsa-staining slide	Positive	Positive	-	34.24	Positive	28.02	Positive
**13**	ANCH-01	Giemsa-staining slide	Positive	Positive	-	34.83	Positive	25.31	Positive
**14**	ANCH-10	Giemsa-staining slide	Positive	Positive	-	39.01	Negative	25.79	Positive
**15**	Araujo Quispe	Giemsa-staining slide	Positive	Positive	-	34.09	Positive	27.18	Positive
**16**	CN-01	Giemsa-staining slide	Positive	Positive	-	33.28	Positive	28.38	Positive
**17**	CN-24	Giemsa-staining slide	Positive	Positive	-	0.00	Negative	32.43	Positive

**18**	CORAC-01	Giemsa-staining slide	Positive	Positive	-	29.81	Positive	26.19	Positive
**19**	CORAC-02	Giemsa-staining slide	Positive	Positive	-	32.76	Positive	23.88	Positive
**20**	CORAC-07 (1)	Giemsa-staining slide	Positive	Positive	-	27.94	Positive	22.63	Positive
**21**	CORAC-08	Giemsa-staining slide	Positive	Positive	-	0.00	Negative	21.85	Positive
**22**	HAH-10 (1)	Giemsa-staining slide	Positive	Positive	-	35.18	Negative	35.83	Positive
**23**	HAH-11	Giemsa-staining slide	Positive	Positive	-	0.00	Negative	33.59	Positive
**24**	HAH-16 (1)	Giemsa-staining slide	Positive	Positive	-	30.09	Positive	30.69	Positive
**25**	HAH-20	Giemsa-staining slide	Positive	Positive	-	32.57	Positive	31.83	Positive
**26**	HAH-22 (2)	Giemsa-staining slide	Positive	Positive	-	33.55	Positive	31.67	Positive
**27**	HAH-23 (1)	Giemsa-staining slide	Positive	Positive	-	32.11	Positive	29.82	Positive
**28**	HAH-24 (1)	Giemsa-staining slide	Positive	Positive	-	31.45	Positive	29.76	Positive
**29**	HAH-24 (2)	Giemsa-staining slide	Positive	Positive	-	33.83	Positive	32.90	Positive
**30**	HAH-27 (1)	Giemsa-staining slide	Positive	Positive	-	27.06	Positive	30.94	Positive
**31**	HAH-27 (2)	Giemsa-staining slide	Positive	Positive	-	33.65	Positive	30.14	Positive
**32**	HAH-30	Giemsa-staining slide	Positive	Positive	-	0.00	Negative	28.68	Positive
**33**	HAH-31	Giemsa-staining slide	Positive	Positive	-	0.00	Negative	32.77	Positive
**34**	HAH-32	Giemsa-staining slide	Positive	Positive	-	0.00	Negative	23.32	Positive
**35**	HAH-39	Giemsa-staining slide	Positive	Positive	-	32.97	Positive	29.48	Positive
**36**	HAH-40	Giemsa-staining slide	Positive	Positive	-	35.16	Positive	31.29	Positive
**37**	HAH-41	Giemsa-staining slide	Positive	Positive	-	35.39	Positive	28.27	Positive

**38**	HRA-01 (1)	Giemsa-staining slide	Positive	Positive	-	-	Negative	26.16	Positive
**39**	HRA-01 (2)	Giemsa-staining slide	Positive	Positive	-	31.51	Positive	35.92	Negative
**40**	HRA-02	Giemsa-staining slide	Positive	Positive	-	0.00	Negative	26.53	Positive
**41**	HRA-03	Giemsa-staining slide	Positive	Positive	-	0.00	Negative	30.44	Positive
**42**	HRA-26	Giemsa-staining slide	Positive	Positive	-	-	Negative	24.43	Positive
**43**	HRA-34	Giemsa-staining slide	Positive	Positive	-	30.95	Positive	29.04	Positive
**44**	HRA-43	Giemsa-staining slide	Positive	Positive	-	35.54	Positive	25.23	Positive
**45**	HRA-57	Giemsa-staining slide	Positive	Positive	-	0.00	Negative	26.05	Positive
**46**	HRA-62	Giemsa-staining slide	Positive	Positive	-	33.95	Positive	30.54	Positive
**47**	HRA-65	Giemsa-staining slide	Positive	Positive	-	33.44	Positive	24.70	Positive
**48**	HSF-01	Giemsa-staining slide	Positive	Positive	-	31.36	Positive	30.58	Positive
**49**	HSF-02	Giemsa-staining slide	Positive	Positive	-	33.62	Positive	37.47	Negative
**50**	HSF-04	Giemsa-staining slide	Positive	Positive	-	0.00	Negative	24.79	Positive
**51**	HSF-06 (1)	Giemsa-staining slide	Positive	Positive	-	-	Negative	35.13	Negative
**52**	HSF-06 (2)	Giemsa-staining slide	Positive	Positive	-	37.74	Negative	30.25	Positive
**53**	HSF-08	Giemsa-staining slide	Positive	Positive	-	34.83	Positive	35.68	Negative
**54**	HSF-10	Giemsa-staining slide	Positive	Positive	-	-	Negative	34.34	Positive
**55**	LL-06 (1)	Giemsa-staining slide	Positive	Positive	-	38.81	Negative	29.49	Positive
**56**	LL-16	Giemsa-staining slide	Positive	Positive	-	29.50	Positive	28.79	Positive
**57**	LL-19	Giemsa-staining slide	Positive	Positive	-	0.00	Negative	32.59	Positive

**58**	LL-20	Giemsa-staining slide	Positive	Positive	-	34.68	Positive	35.33	Negative
**59**	LL-23	Giemsa-staining slide	Positive	Positive	-	30.06	Positive	25.30	Positive
**60**	LL-29	Giemsa-staining slide	Positive	Positive	-	30.53	Positive	31.27	Positive
**61**	LL-30	Giemsa-staining slide	Positive	Positive	-	31.30	Positive	27.64	Positive
**62**	LL-47	Giemsa-staining slide	Positive	Positive	-	33.24	Positive	27.98	Positive
**63**	LL-48	Giemsa-staining slide	Positive	Positive	-	34.09	Positive	24.17	Positive
**64**	LL-52	Giemsa-staining slide	Positive	Positive	-	31.87	Positive	27.45	Positive
**65**	LL-64	Giemsa-staining slide	Positive	Positive	-	34.62	Positive	31.87	Positive
**66**	LL-70	Giemsa-staining slide	Positive	Positive	-	33.72	Positive	31.35	Positive
**67**	LL-73	Giemsa-staining slide	Positive	Positive	-	0.00	Negative	35.87	Positive
**68**	LL-75	Giemsa-staining slide	Positive	Positive	-	34.97	Positive	26.50	Positive
**69**	LLOA-04	Giemsa-staining slide	Positive	Positive	-	0.00	Negative	31.38	Positive
**70**	LLOA-81	Giemsa-staining slide	Positive	Positive	-	33.91	Positive	19.57	Positive
**71**	LSM-7-01	Giemsa-staining slide	Positive	Positive	-	0.00	Negative	23.06	Positive
**72**	NN-26	Giemsa-staining slide	Positive	Positive	-	33.37	Positive	31.69	Positive
**73**	PP-01	Giemsa-staining slide	Positive	Positive	-	0.00	Negative	31.44	Positive
**74**	PP-03 (2)	Giemsa-staining slide	Positive	Positive	-	34.37	Positive	26.92	Positive
**75**	SE-01 (1)	Giemsa-staining slide	Positive	Positive	-	0.00	Negative	35.05	Positive
**76**	SE-01 (2)	Giemsa-staining slide	Positive	Positive	-	-	Negative	26.03	Positive
**77**	SF-01	Giemsa-staining slide	Positive	Positive	-	28.99	Positive	26.38	Positive

**78**	SF-02	Giemsa-staining slide	Positive	Positive	-	0.00	Negative	30.58	Positive
**79**	SF-03	Giemsa-staining slide	Positive	Positive	-	0.00	Negative	25.16	Positive
**80**	SF-04	Giemsa-staining slide	Positive	Positive	-	28.78	Positive	28.84	Positive
**81**	SF-16	Giemsa-staining slide	Positive	Positive	-	36.45	Negative	26.22	Positive
**82**	SF-18	Giemsa-staining slide	Positive	Positive	-	29.13	Positive	28.11	Positive
**83**	SF-19	Giemsa-staining slide	Positive	Positive	-	35.35	Negative	29.49	Positive
**84**	SF-28	Giemsa-staining slide	Positive	Positive	-	28.22	Positive	28.02	Positive
**85**	SF-30	Giemsa-staining slide	Positive	Positive	-	29.95	Positive	25.85	Positive
**86**	SF-31	Giemsa-staining slide	Positive	Positive	-	0.00	Negative	32.44	Positive
**87**	SF-43	Giemsa-staining slide	Positive	Positive	-	35.69	Negative	25.16	Positive
**88**	SF-47	Giemsa-staining slide	Positive	Positive	-	0.00	Negative	31.70	Positive
**89**	SJS-03 (1)	Giemsa-staining slide	Positive	Positive	-	36.46	Negative	29.07	Positive
**90**	SJS-03 (2)	Giemsa-staining slide	Positive	Positive	-	0.00	Negative	33.41	Positive
**91**	SJS-04	Giemsa-staining slide	Positive	Positive	-	34.67	Positive	28.43	Positive
**92**	SM-02 (1)	Giemsa-staining slide	Positive	Positive	-	0.00	Negative	33.20	Positive
**93**	SM-03 (1)	Giemsa-staining slide	Positive	Positive	-	34.39	Positive	29.77	Positive
**94**	SM-03 (2)	Giemsa-staining slide	Positive	Positive	-	35.99	Negative	25.54	Positive
**95**	SM-07	Giemsa-staining slide	Positive	Positive	-	32.23	Positive	21.20	Positive
**96**	SM-08	Giemsa-staining slide	Positive	Positive	-	31.13	Positive	30.43	Positive
**97**	SM-10	Giemsa-staining slide	Positive	Positive	-	36.83	Negative	29.54	Positive

**98**	SM-19	Giemsa-staining slide	Positive	Positive	-	34.03	Positive	28.28	Positive
**99**	SM-21	Giemsa-staining slide	Positive	Positive	-	27.97	Positive	29.93	Positive
**100**	SR-01 (1)	Giemsa-staining slide	Positive	Positive	-	0.00	Negative	33.85	Positive
**101**	SR-03 (1)	Giemsa-staining slide	Positive	Positive	-	33.48	Positive	25.20	Positive
**102**	SR-03 (2)	Giemsa-staining slide	Positive	Positive	-	0.00	Negative	28.42	Positive
**103**	SR-04 (1)	Giemsa-staining slide	Positive	Positive	-	0.00	Negative	29.60	Positive
**104**	SR-04 (2)	Giemsa-staining slide	Positive	Positive	-	0.00	Negative	21.92	Positive
**105**	SR-15	Giemsa-staining slide	Positive	Positive	-	33.35	Positive	31.48	Positive
**106**	SR-18	Giemsa-staining slide	Positive	Positive	-	32.68	Positive	38.40	Negative
**107**	SV-02	Giemsa-staining slide	Positive	Positive	-	35.69	Negative	26.90	Positive
**108**	SV-19	Giemsa-staining slide	Positive	Positive	-	37.81	Negative	32.07	Positive
**109**	SV-22	Giemsa-staining slide	Positive	Positive	-	0.00	Negative	21.75	Positive
**110**	SV-23	Giemsa-staining slide	Positive	Positive	-	0.00	Negative	27.46	Positive
**111**	TB-01	Giemsa-staining slide	Positive	Positive	-	28.56	Positive	31.28	Positive
**112**	TB-02	Giemsa-staining slide	Positive	Positive	-	0.00	Negative	27.31	Positive
**113**	TB-03	Giemsa-staining slide	Positive	Positive	-	0.00	Negative	33.07	Positive
**114**	35 SAA 2013	Stainless steel lancet	Positive	Positive	Positive	28.78	Positive	18.05	Positive
**115**	14 FNJ 2013	Stainless steel lancet	Positive	Positive	Positive	28.68	Positive	18.17	Positive
**116**	10 ORA 2018	Stainless steel lancet	Positive	Positive	Positive	34.88	Positive	18.41	Positive
**117**	51 MZJ 2013	Stainless steel lancet	Positive	Positive	Positive	29.05	Positive	18.58	Positive

**118**	4 M	Stainless steel lancet	Positive	Positive	Positive	25.64	Positive	19.10	Positive
**119**	34 SSL 2016	Stainless steel lancet	Positive	Positive	Positive	39.33	Negative	19.12	Positive
**120**	41 VRW 2014	Stainless steel lancet	Positive	Positive	-	32.25	Positive	19.65	Positive
**121**	14 FVL 2011	Stainless steel lancet	Positive	Positive	Positive	25.72	Positive	20.04	Positive
**122**	49 ACM 2016	Stainless steel lancet	Positive	Positive	Positive	0.00	Negative	20.37	Positive
**123**	22 MEM 2011	Stainless steel lancet	Positive	Positive	Positive	26.72	Positive	20.72	Positive
**124**	30 RRM 2014	Stainless steel lancet	Positive	Positive	-	0.00	Negative	20.72	Positive
**125**	8 AGJ 2014	Stainless steel lancet	Positive	Positive	-	33.22	Positive	20.81	Positive
**126**	23 GCJ 2015	Stainless steel lancet	Positive	Positive	-	0.00	Negative	20.97	Positive
**127**	33 RRM 2016	Stainless steel lancet	Positive	Positive	Positive	0.00	Negative	21.22	Positive
**128**	45 VAJ 2016	Stainless steel lancet	Positive	Positive	Positive	28.31	Positive	21.36	Positive
**129**	47 AAA 2014	Stainless steel lancet	Positive	Positive	-	0.00	Negative	21.61	Positive
**130**	18 PSM 2014	Stainless steel lancet	Positive	Positive	-	32.60	Positive	21.66	Positive
**131**	36 RGJ 2015	Stainless steel lancet	Positive	Positive	Positive	34.80	Positive	21.85	Positive
**132**	32 RRM 2014	Stainless steel lancet	Positive	Positive	-	35.57	Negative	21.89	Positive
**133**	46 RCJ 2014	Stainless steel lancet	Positive	Positive	-	0.00	Negative	21.90	Positive
**134**	21 MGR 2015	Stainless steel lancet	Positive	Positive	-	39.81	Negative	21.97	Positive
**135**	5 RSR 2010	Stainless steel lancet	Positive	Positive	Positive	30.13	Positive	22.03	Positive
**136**	35 RNN 2011	Stainless steel lancet	Positive	Positive	Positive	26.73	Positive	22.11	Positive
**137**	16 GTM 2013	Stainless steel lancet	Positive	Positive	Positive	28.14	Positive	22.11	Positive

**138**	2 GVM 2015	Stainless steel lancet	Positive	Positive	Positive	28.65	Positive	22.15	Positive
**139**	32 RDM 2013	Stainless steel lancet	Positive	Positive	Positive	36.09	Negative	22.48	Positive
**140**	8 M	Stainless steel lancet	Positive	Positive	Positive	24.47	Positive	22.66	Positive
**141**	25 GCJ 2015	Stainless steel lancet	Positive	Positive	-	39.71	Negative	22.70	Positive
**142**	7 DMW 2018	Stainless steel lancet	Positive	Positive	-	37.17	Negative	22.71	Positive
**143**	20 MAW 2015	Stainless steel lancet	Positive	Positive	-	0.00	Negative	22.92	Positive
**144**	60 LCA 2014	Stainless steel lancet	Positive	Positive	-	37.42	Negative	23.02	Positive
**145**	31 FTJ 2013	Stainless steel lancet	Positive	Positive	Positive	27.90	Positive	23.05	Positive
**146**	7 CLJ 2015	Stainless steel lancet	Positive	Positive	Positive	34.20	Positive	23.07	Positive
**147**	18RNJ 2013	Stainless steel lancet	Positive	Positive	-	22.31	Positive	23.18	Positive
**148**	24 GCJ 2015	Stainless steel lancet	Positive	Positive	-	35.65	Negative	23.25	Positive
**149**	9 M	Stainless steel lancet	Positive	Positive	Positive	26.09	Positive	23.29	Positive
**150**	9 CO 2015	Stainless steel lancet	Positive	Positive	Positive	22.74	Positive	23.54	Positive
**151**	41 ZGE 2013	Stainless steel lancet	Positive	Positive	Positive	30.32	Positive	23.62	Positive
**152**	14 MMJ 2015	Stainless steel lancet	Positive	Positive	-	0.00	Negative	23.78	Positive
**153**	40 SFH 2013	Stainless steel lancet	Positive	Positive	Positive	32.80	Positive	23.80	Positive
**154**	39 BSE 2013	Stainless steel lancet	Positive	Positive	-	28.44	Positive	23.95	Positive
**155**	14 RHE 2014	Stainless steel lancet	Positive	Positive	-	0.00	Negative	23.97	Positive
**156**	35 SPJ 2016	Stainless steel lancet	Positive	Positive	Positive	37.75	Negative	24.10	Positive
**157**	52 JDCM 2016	Stainless steel lancet	Positive	Positive	Positive	30.52	Positive	24.13	Positive

**158**	42 VDPO 2014	Stainless steel lancet	Positive	Positive	-	35.01	Negative	24.69	Positive
**159**	6ESP 2010	Stainless steel lancet	Positive	Positive	Positive	32.34	Positive	24.70	Positive
**160**	29 MCE 2013	Stainless steel lancet	Positive	Positive	Positive	36.38	Negative	25.21	Positive
**161**	47 BCA 2013	Stainless steel lancet	Positive	Positive	2	29.89	Positive	25.30	Positive
**162**	18 MRJ 2010	Stainless steel lancet	Positive	Positive	-	32.02	Positive	25.34	Positive
**163**	4 LLJ 2010	Stainless steel lancet	Positive	Positive	Positive	26.75	Positive	25.42	Positive
**164**	46 MAJ 2016	Stainless steel lancet	Positive	Positive	Positive	27.75	Positive	25.65	Positive
**165**	22 M	Stainless steel lancet	Positive	Positive	-	29.51	Positive	25.67	Positive
**166**	36 RGV 2013	Stainless steel lancet	Positive	Positive	Positive	30.79	Positive	25.75	Positive
**167**	37 HMM 2015	Stainless steel lancet	Positive	Positive	-	0.00	Negative	25.89	Positive
**168**	8 ISL 2010	Stainless steel lancet	Positive	Positive	Positive	25.40	Positive	25.90	Positive
**169**	33 QYA 2014	Stainless steel lancet	Positive	Positive	-	0.00	Negative	25.92	Positive
**170**	6 CNA 2018	Stainless steel lancet	Positive	Positive	Positive	35.31	Negative	26.08	Positive
**171**	38 ZAM 2014	Stainless steel lancet	Positive	Positive	-	0.00	Negative	26.10	Positive
**172**	35 SQJ 2014	Stainless steel lancet	Positive	Positive	-	36.23	Negative	26.17	Positive
**173**	22 RCN 2013	Stainless steel lancet	Positive	Positive	Positive	0.00	Negative	26.42	Positive
**174**	8 NJV 2018	Stainless steel lancet	Positive	Positive	-	29.70	Positive	26.62	Positive
**175**	20 FGM 2011	Stainless steel lancet	Positive	Positive	-	27.20	Positive	26.93	Positive
**176**	30 PRJ 2015	Stainless steel lancet	Positive	Positive	-	37.25	Negative	26.93	Positive
**177**	27 M	Stainless steel lancet	Positive	Positive	Positive	29.56	Positive	27.09	Positive

**178**	17 MDS 2015	Stainless steel lancet	Positive	Positive	Positive	24.11	Positive	27.19	Positive
**179**	19 TFM 2011	Stainless steel lancet	Positive	Positive	-	27.12	Positive	27.38	Positive
**180**	15 BSO 2013	Stainless steel lancet	Positive	Positive	Negative	25.78	Positive	27.38	Positive
**181**	11 HGJ 2014	Stainless steel lancet	Positive	Positive	Negative	0.00	Negative	27.61	Positive
**182**	2 FPG 2014	Stainless steel lancet	Positive	Positive	-	31.30	Positive	27.67	Positive
**183**	17 AAI 2010	Stainless steel lancet	Positive	Positive	-	30.37	Positive	27.89	Positive
**184**	9 TFM 2011	Stainless steel lancet	Positive	Positive	-	25.57	Positive	27.97	Positive
**185**	13 GML 2010	Stainless steel lancet	Positive	Positive	Positive	29.02	Positive	28.04	Positive
**186**	1EML 2014	Stainless steel lancet	Positive	Positive	-	35.29	Negative	28.15	Positive
**187**	19 MYV 2014	Stainless steel lancet	Positive	Positive	-	34.88	Positive	28.16	Positive
**188**	35 LTA 2015	Stainless steel lancet	Positive	Positive	Positive	0.00	Negative	28.31	Positive
**189**	28 M	Stainless steel lancet	Positive	Positive	-	32.54	Positive	28.33	Positive
**190**	31 RRM 2014	Stainless steel lancet	Positive	Positive	-	37.35	Negative	28.34	Positive
**191**	17 M	Stainless steel lancet	Positive	Positive	-	26.16	Positive	28.48	Positive
**192**	27 SGJ 2015	Stainless steel lancet	Positive	Positive	Positive	30.02	Positive	28.87	Positive
**193**	15 FGJ 2011	Stainless steel lancet	Positive	Positive	-	25.96	Positive	28.91	Positive
**194**	43 CHE 2015	Stainless steel lancet	Positive	Positive	-	0.00	Negative	28.96	Positive
**195**	30 CBM 2013	Stainless steel lancet	Positive	Positive	Positive	33.02	Positive	29.01	Positive
**196**	22 CPA 2015	Stainless steel lancet	Positive	Positive	-	35.48	Negative	29.14	Positive
**197**	29 SFM 2014	Stainless steel lancet	Positive	Positive	-	36.55	Negative	29.15	Positive

**198**	36 RBA 2014	Stainless steel lancet	Positive	Positive	-	39.35	Negative	29.24	Positive
**199**	6 CQM 2014	Stainless steel lancet	Positive	Positive	-	31.60	Positive	29.84	Positive
**200**	28 BAJ 2016	Stainless steel lancet	Positive	Positive	Positive	0.00	Negative	29.92	Positive
**201**	11 BBL 2013	Stainless steel lancet	Positive	Positive	-	24.98	Positive	29.97	Positive
**202**	21 QVJ 2013	Stainless steel lancet	Positive	Positive	Positive	29.39	Positive	30.14	Positive
**203**	18 EUA 2011	Stainless steel lancet	Positive	Negative	Positive	28.33	Positive	30.19	Positive
**204**	21 FVH 2011	Stainless steel lancet	Positive	Positive	Positive	23.48	Positive	30.27	Positive
**205**	12 GBK 2015	Stainless steel lancet	Positive	Positive	-	0.00	Negative	30.64	Positive
**206**	36 SPJ 2016	Stainless steel lancet	Positive	Positive	Positive	39.65	Negative	31.14	Positive
**207**	13 PGN 2015	Stainless steel lancet	Positive	Positive	-	0.00	Negative	32.22	Positive
**208**	2 AMT 2011	Stainless steel lancet	Positive	Positive	-	24.36	Positive	32.35	Positive
**209**	25 CCF 2010	Stainless steel lancet	Positive	Positive	Negative	33.98	Positive	32.38	Positive
**210**	17 TEH 2013	Stainless steel lancet	Positive	Positive	Positive	27.12	Positive	32.59	Positive
**211**	38 PRG 2013	Stainless steel lancet	Positive	Positive	Positive	34.20	Positive	33.07	Positive
**212**	50 FJA 2013	Stainless steel lancet	Positive	-	Positive	36.28	Negative	33.17	Positive
**213**	9 NJV 2018	Stainless steel lancet	Positive	Positive	-	39.22	Negative	33.60	Positive
**214**	17 FLJ 2016	Stainless steel lancet	Positive	Positive	-	0.00	Negative	35.55	Positive
**215**	62 MBB 2014	Stainless steel lancet	Positive	Positive	-	31.09	Positive	35.95	Positive
**216**	5 BGL 2013	Stainless steel lancet	Positive	Positive	-	27.32	Positive	36.50	Negative
**217**	9 HJM 2013	Stainless steel lancet	Positive	Negative	Positive	30.77	Positive	38.94	Negative

**218**	26 M	Stainless steel lancet	Positive	Positive	-	32.86	Positive	39.26	Negative
**219**	3 CDJ 2015	Stainless steel lancet	Positive	Positive	-	34.66	Positive	40.76	Negative
**220**	3 M	Stainless steel lancet	Positive	Positive	Positive	32.84	Positive	0.00	Negative
**221**	7 M	Stainless steel lancet	Positive	Negative	Positive	27.21	Positive	0.00	Negative
**222**	10 M	Stainless steel lancet	Positive	Negative	Positive	31.73	Positive	0.00	Negative
**223**	11 M	Stainless steel lancet	Positive	Negative	Positive	28.85	Positive	0.00	Negative
**224**	12 M	Stainless steel lancet	Positive	Negative	Positive	30.28	Positive	0.00	Negative
**225**	24 M	Stainless steel lancet	Positive	Positive	Positive	30.76	Positive	0.00	Negative
**226**	L1	Stainless steel lancet	Negative	Negative	-	1.00	Positive	0.00	Negative
**227**	L2	Stainless steel lancet	Negative	Negative	-	1.00	Positive	0.00	Negative
**228**	L3	Stainless steel lancet	Negative	Negative	-	1.00	Positive	0.00	Negative
**229**	L4	Stainless steel lancet	Negative	Negative	-	1.00	Positive	0.00	Negative
**230**	L5	Stainless steel lancet	Negative	Negative	-	1.00	Positive	0.00	Negative
**231**	L6	Stainless steel lancet	Negative	Negative	-	1.00	Positive	0.00	Negative
**232**	L7	Stainless steel lancet	Negative	Negative	-	1.00	Positive	0.00	Negative
**233**	L8	Stainless steel lancet	Negative	Negative	-	1.00	Positive	0.00	Negative
**234**	L9	Stainless steel lancet	Negative	Negative	-	1.00	Positive	0.00	Negative
**235**	L10	Stainless steel lancet	Negative	Negative	-	1.00	Positive	0.00	Negative
**236**	L11	Stainless steel lancet	Negative	Negative	-	1.00	Positive	0.00	Negative
**237**	L12	Stainless steel lancet	Negative	Negative	-	1.00	Positive	0.00	Negative

**238**	L13	Stainless steel lancet	Negative	Negative	-	1.00	Positive	0.00	Negative
**239**	L14	Stainless steel lancet	Negative	Negative	-	1.00	Positive	0.00	Negative
**240**	L15	Stainless steel lancet	Negative	Negative	-	1.00	Positive	0.00	Negative
**241**	L16	Stainless steel lancet	Negative	Negative	-	1.00	Positive	0.00	Negative
**242**	L17	Stainless steel lancet	Negative	Negative	-	1.00	Positive	0.00	Negative
**243**	L18	Stainless steel lancet	Negative	Negative	-	1.00	Positive	0.00	Negative
**244**	L19	Stainless steel lancet	Negative	Negative	-	1.00	Positive	0.00	Negative
**245**	L20	Stainless steel lancet	Negative	Negative	-	1.00	Positive	0.00	Negative
**246**	L21	Stainless steel lancet	Negative	Negative	-	1.00	Positive	0.00	Negative
**247**	L22	Stainless steel lancet	Negative	Negative	-	1.00	Positive	0.00	Negative
**248**	L23	Stainless steel lancet	Negative	Negative	-	1.00	Positive	0.00	Negative
**249**	L24	Stainless steel lancet	Negative	Negative	-	1.00	Positive	0.00	Negative
**250**	L25	Stainless steel lancet	Negative	Negative	-	1.00	Positive	0.00	Negative
**251**	L26	Stainless steel lancet	Negative	Negative	-	1.00	Positive	0.00	Negative
**252**	L27	Stainless steel lancet	Negative	Negative	-	1.00	Positive	0.00	Negative
**253**	L28	Stainless steel lancet	Negative	Negative	-	1.00	Positive	0.00	Negative
**254**	L29	Stainless steel lancet	Negative	Negative	-	1.00	Positive	0.00	Negative
**255**	L30	Stainless steel lancet	Negative	Negative	-	1.00	Positive	0.00	Negative
**256**	L31	Stainless steel lancet	Negative	Negative	-	1.00	Positive	0.00	Negative
**257**	L32	Stainless steel lancet	Negative	Negative	-	1.00	Positive	0.00	Negative

**258**	L33	Stainless steel lancet	Negative	Negative	-	1.00	Positive	0.00	Negative
**259**	L34	Stainless steel lancet	Negative	Negative	-	1.00	Positive	0.00	Negative
**260**	L35	Stainless steel lancet	Negative	Negative	-	1.00	Positive	0.00	Negative
**261**	L36	Stainless steel lancet	Negative	Negative	-	1.00	Positive	0.00	Negative
**262**	L37	Stainless steel lancet	Negative	Negative	-	1.00	Positive	0.00	Negative
**263**	L38	Stainless steel lancet	Negative	Negative	-	1.00	Positive	0.00	Negative
**264**	L39	Stainless steel lancet	Negative	Negative	-	1.00	Positive	0.00	Negative
**265**	L40	Stainless steel lancet	Negative	Negative	-	1.00	Positive	0.00	Negative
**266**	L41	Stainless steel lancet	Negative	Negative	-	1.00	Positive	0.00	Negative
**267**	L42	Stainless steel lancet	Negative	Negative	-	1.00	Positive	0.00	Negative
**268**	L43	Stainless steel lancet	Negative	Negative	-	1.00	Positive	0.00	Negative
**269**	L44	Stainless steel lancet	Negative	Negative	-	1.00	Positive	0.00	Negative
**270**	L45	Stainless steel lancet	Negative	Negative	-	1.00	Positive	0.00	Negative
**271**	L46	Stainless steel lancet	Negative	Negative	-	1.00	Positive	0.00	Negative
**272**	L47	Stainless steel lancet	Negative	Negative	-	1.00	Positive	0.00	Negative
**273**	L48	Stainless steel lancet	Negative	Negative	-	1.00	Positive	0.00	Negative
**274**	L49	Stainless steel lancet	Negative	Negative	-	1.00	Positive	0.00	Negative
**275**	L50	Stainless steel lancet	Negative	Negative	-	1.00	Positive	0.00	Negative
**276**	L51	Stainless steel lancet	Negative	Negative	-	1.00	Positive	0.00	Negative
**277**	L52	Stainless steel lancet	Negative	Negative	-	1.00	Positive	0.00	Negative

**278**	L53	Stainless steel lancet	Negative	Negative	-	1.00	Positive	0.00	Negative
**279**	L54	Stainless steel lancet	Negative	Negative	-	1.00	Positive	0.00	Negative
**280**	L55	Stainless steel lancet	Negative	Negative	-	1.00	Positive	0.00	Negative
**281**	L56	Stainless steel lancet	Negative	Negative	-	1.00	Positive	0.00	Negative
**282**	L57	Stainless steel lancet	Negative	Negative	-	1.00	Positive	0.00	Negative
**283**	L58	Stainless steel lancet	Negative	Negative	-	1.00	Positive	0.00	Negative
**284**	L59	Stainless steel lancet	Negative	Negative	-	1.00	Positive	0.00	Negative
**285**	L60	Stainless steel lancet	Negative	Negative	-	1.00	Positive	0.00	Negative
**286**	L61	Stainless steel lancet	Negative	Negative	-	1.00	Positive	0.00	Negative
**287**	L62	Stainless steel lancet	Negative	Negative	-	1.00	Positive	0.00	Negative
**288**	L63	Stainless steel lancet	Negative	Negative	-	1.00	Positive	0.00	Negative
**289**	L64	Stainless steel lancet	Negative	Negative	-	1.00	Positive	0.00	Negative
**290**	L65	Stainless steel lancet	Negative	Negative	-	1.00	Positive	0.00	Negative
**291**	L66	Stainless steel lancet	Negative	Negative	-	1.00	Positive	0.00	Negative

DME: Direct Microscopic Examination; Ct: Cycle of threshold
