# Supplementary material for: Validation of a Real-Time PCR for the Diagnosis of Leishmania Species Using the Hsp20 Gene
Source: Trop Med Infect Dis. 2025 May 1;10(5):121. doi: 10.3390/tropicalmed10050121 (PMC12115578; doi:10.3390/tropicalmed10050121)
Supplement: Supplementary file 1 [file tropicalmed-10-00121-s001.zip › Supplementary material Table S2.pdf]

Table S2. Characteristics of the clinical samples of confirmed patients included in the study evaluated using a marked probe.

N°	Code	Sample	Gold Standard result	DME Result	Culture result	Ct_RNAse P	RNAseP result	Ct_Hsp20	Hsp20 result
**1**	10 M	Stainless steel lancet	Positive	Negative	Positive	31.73	Positive	0.00	Negative
**2**	10 ORA 2018	Stainless steel lancet	Positive	Positive	Positive	34.88	Positive	18.40	Positive
**3**	11 BBL 2013	Stainless steel lancet	Positive	Positive	Negative	24.98	Positive	30.00	Positive
**4**	11 HGJ 2014	Stainless steel lancet	Positive	Positive	Negative	0.00	Negative	27.60	Positive
**5**	11 M	Stainless steel lancet	Positive	Negative	Positive	28.85	Positive	0.00	Negative
**6**	11 RTN 2016	Stainless steel lancet	Positive	Positive	Negative	0.00	Negative	0.00	Negative
**7**	12 GBK 2015	Stainless steel lancet	Positive	Positive	Negative	0.00	Negative	30.60	Positive
**8**	12 M	Stainless steel lancet	Positive	Negative	Positive	30.28	Positive	0.00	Negative
**9**	12 RTN 2016	Stainless steel lancet	Positive	Positive	Negative	39.04	Negative	0.00	Negative
**10**	13 GML 2010	Stainless steel lancet	Positive	Positive	Positive	29.02	Positive	28.00	Positive
**11**	13 PGN 2015	Stainless steel lancet	Positive	Positive	Negative	0.00	Negative	32.20	Positive
**12**	14 FNJ 2013	Stainless steel lancet	Positive	Positive	Positive	28.68	Positive	18.20	Positive
**13**	14 FVL 2011	Stainless steel lancet	Positive	Positive	Positive	25.72	Positive	20.00	Positive
**14**	14 MMJ 2015	Stainless steel lancet	Positive	Positive	Negative	0.00	Negative	23.80	Positive
**15**	14 RHE 2014	Stainless steel lancet	Positive	Positive	Negative	0.00	Negative	24.00	Positive
**16**	15 BSO 2013	Stainless steel lancet	Positive	Positive	Negative	25.78	Positive	27.40	Positive
**17**	15 FGJ 2011	Stainless steel lancet	Positive	Positive	Negative	25.96	Positive	28.90	Positive

**18**	16 GTM 2013	Stainless steel lancet	Positive	Positive	Positive	28.14	Positive	22.10	Positive
**19**	17 AAI 2010	Stainless steel lancet	Positive	Positive	Negative	30.37	Positive	27.90	Positive
**20**	17 FLJ 2016	Stainless steel lancet	Positive	Positive	Negative	0.00	Negative	35.60	Positive
**21**	17 GCB 2014	Stainless steel lancet	Positive	Positive	Negative	0.00	Negative	0.00	Negative
**22**	17 M	Stainless steel lancet	Positive	Positive	Negative	26.16	Positive	28.50	Positive
**23**	17 MDS 2015	Stainless steel lancet	Positive	Positive	Positive	24.11	Positive	27.20	Positive
**24**	18 JNA 2016	Stainless steel lancet	Positive	Positive	Negative	0.00	Negative	0.00	Negative
**25**	18 M	Stainless steel lancet	Positive	Positive	Positive	37.20	Negative	0.00	Negative
**26**	22 CGJ 2016	Stainless steel lancet	Positive	Positive	Negative	0.00	Negative	43.10	Negative
**27**	24 M	Stainless steel lancet	Positive	Positive	Positive	30.76	Positive	0.00	Negative
**28**	25 GHE 2016	Stainless steel lancet	Positive	Positive	Positive	0.00	Negative	0.00	Negative
**29**	25 LSG 2014	Stainless steel lancet	Positive	Positive	Negative	0.00	Negative	0.00	Negative
**30**	26 M	Stainless steel lancet	Positive	Positive	Negative	32.86	Positive	39.30	Negative
**31**	3 CDJ 2015	Stainless steel lancet	Positive	Positive	Negative	34.66	Positive	40.80	Negative
**32**	3 M	Stainless steel lancet	Positive	Positive	Positive	32.84	Positive	0.00	Negative
**33**	3 STR 2010	Stainless steel lancet	Positive	Positive	Negative	0.00	Negative	0.00	Negative
**34**	30 M	Stainless steel lancet	Positive	Positive	Negative	0.00	Negative	0.00	Negative
**35**	32 RRM 2016	Stainless steel lancet	Positive	Positive	Positive	0.00	Negative	0.00	Negative
**36**	39 FGC 2015	Stainless steel lancet	Positive	Positive	Negative	39.36	Negative	0.00	Negative
**37**	L1	Stainless steel lancet	Negative	Negative	Negative	0.00	Positive	0.00	Negative

**38**	L2	Stainless steel lancet	Negative	Negative	Negative	0.00	Positive	0.00	Negative
**39**	L3	Stainless steel lancet	Negative	Negative	Negative	0.00	Positive	0.00	Negative
**40**	L4	Stainless steel lancet	Negative	Negative	Negative	0.00	Positive	0.00	Negative
**41**	L5	Stainless steel lancet	Negative	Negative	Negative	0.00	Positive	0.00	Negative
**42**	L6	Stainless steel lancet	Negative	Negative	Negative	0.00	Positive	0.00	Negative
**43**	L7	Stainless steel lancet	Negative	Negative	Negative	0.00	Positive	0.00	Negative
**44**	L8	Stainless steel lancet	Negative	Negative	Negative	0.00	Positive	0.00	Negative
**45**	L9	Stainless steel lancet	Negative	Negative	Negative	0.00	Positive	0.00	Negative
**46**	L10	Stainless steel lancet	Negative	Negative	Negative	0.00	Positive	0.00	Negative
**47**	L11	Stainless steel lancet	Negative	Negative	Negative	0.00	Positive	0.00	Negative
**48**	L12	Stainless steel lancet	Negative	Negative	Negative	0.00	Positive	0.00	Negative
**49**	L13	Stainless steel lancet	Negative	Negative	Negative	0.00	Positive	0.00	Negative
**50**	L14	Stainless steel lancet	Negative	Negative	Negative	0.00	Positive	0.00	Negative
**51**	L15	Stainless steel lancet	Negative	Negative	Negative	0.00	Positive	0.00	Negative
**52**	L16	Stainless steel lancet	Negative	Negative	Negative	0.00	Positive	0.00	Negative
**53**	L17	Stainless steel lancet	Negative	Negative	Negative	0.00	Positive	0.00	Negative
**54**	L18	Stainless steel lancet	Negative	Negative	Negative	0.00	Positive	0.00	Negative
**55**	L19	Stainless steel lancet	Negative	Negative	Negative	0.00	Positive	0.00	Negative
**56**	L20	Stainless steel lancet	Negative	Negative	Negative	0.00	Positive	0.00	Negative
**57**	L21	Stainless steel lancet	Negative	Negative	Negative	0.00	Positive	0.00	Negative

**58**	L22	Stainless steel lancet	Negative	Negative	Negative	0.00	Positive	0.00	Negative
**59**	L23	Stainless steel lancet	Negative	Negative	Negative	0.00	Positive	0.00	Negative
**60**	L24	Stainless steel lancet	Negative	Negative	Negative	0.00	Positive	0.00	Negative
**61**	L25	Stainless steel lancet	Negative	Negative	Negative	0.00	Positive	0.00	Negative
**62**	L26	Stainless steel lancet	Negative	Negative	Negative	0.00	Positive	0.00	Negative
**63**	L27	Stainless steel lancet	Negative	Negative	Negative	0.00	Positive	0.00	Negative
**64**	L28	Stainless steel lancet	Negative	Negative	Negative	0.00	Positive	0.00	Negative
**65**	L29	Stainless steel lancet	Negative	Negative	Negative	0.00	Positive	0.00	Negative
**66**	L30	Stainless steel lancet	Negative	Negative	Negative	0.00	Positive	0.00	Negative
**67**	L31	Stainless steel lancet	Negative	Negative	Negative	0.00	Positive	0.00	Negative
**68**	L32	Stainless steel lancet	Negative	Negative	Negative	0.00	Positive	0.00	Negative
**69**	L33	Stainless steel lancet	Negative	Negative	Negative	0.00	Positive	0.00	Negative
**70**	L34	Stainless steel lancet	Negative	Negative	Negative	0.00	Positive	0.00	Negative
**71**	L35	Stainless steel lancet	Negative	Negative	Negative	0.00	Positive	0.00	Negative
**72**	L36	Stainless steel lancet	Negative	Negative	Negative	0.00	Positive	0.00	Negative
**73**	L37	Stainless steel lancet	Negative	Negative	Negative	0.00	Positive	0.00	Negative
**74**	L38	Stainless steel lancet	Negative	Negative	Negative	0.00	Positive	0.00	Negative
**75**	L39	Stainless steel lancet	Negative	Negative	Negative	0.00	Positive	0.00	Negative
**76**	L40	Stainless steel lancet	Negative	Negative	Negative	0.00	Positive	0.00	Negative
**77**	L41	Stainless steel lancet	Negative	Negative	Negative	0.00	Positive	0.00	Negative

**78**	L42	Stainless steel lancet	Negative	Negative	Negative	0.00	Positive	0.00	Negative
**79**	L43	Stainless steel lancet	Negative	Negative	Negative	0.00	Positive	0.00	Negative
**80**	L44	Stainless steel lancet	Negative	Negative	Negative	0.00	Positive	0.00	Negative
**81**	L45	Stainless steel lancet	Negative	Negative	Negative	0.00	Positive	0.00	Negative
**82**	L46	Stainless steel lancet	Negative	Negative	Negative	0.00	Positive	0.00	Negative
**83**	L47	Stainless steel lancet	Negative	Negative	Negative	0.00	Positive	0.00	Negative
**84**	L48	Stainless steel lancet	Negative	Negative	Negative	0.00	Positive	0.00	Negative
**85**	L49	Stainless steel lancet	Negative	Negative	Negative	0.00	Positive	0.00	Negative
**86**	L50	Stainless steel lancet	Negative	Negative	Negative	0.00	Positive	0.00	Negative
**87**	L51	Stainless steel lancet	Negative	Negative	Negative	0.00	Positive	0.00	Negative
**88**	L52	Stainless steel lancet	Negative	Negative	Negative	0.00	Positive	0.00	Negative
**89**	L53	Stainless steel lancet	Negative	Negative	Negative	0.00	Positive	0.00	Negative
**90**	L54	Stainless steel lancet	Negative	Negative	Negative	0.00	Positive	0.00	Negative
**91**	L55	Stainless steel lancet	Negative	Negative	Negative	0.00	Positive	0.00	Negative
**92**	L56	Stainless steel lancet	Negative	Negative	Negative	0.00	Positive	0.00	Negative
**93**	L57	Stainless steel lancet	Negative	Negative	Negative	0.00	Positive	0.00	Negative
**94**	L58	Stainless steel lancet	Negative	Negative	Negative	0.00	Positive	0.00	Negative
**95**	L59	Stainless steel lancet	Negative	Negative	Negative	0.00	Positive	0.00	Negative
**96**	L60	Stainless steel lancet	Negative	Negative	Negative	0.00	Positive	0.00	Negative
**97**	L61	Stainless steel lancet	Negative	Negative	Negative	0.00	Positive	0.00	Negative

**98**	L62	Stainless steel lancet	Negative	Negative	Negative	0.00	Positive	0.00	Negative
**99**	L63	Stainless steel lancet	Negative	Negative	Negative	0.00	Positive	0.00	Negative
**100**	L64	Stainless steel lancet	Negative	Negative	Negative	0.00	Positive	0.00	Negative
**101**	L65	Stainless steel lancet	Negative	Negative	Negative	0.00	Positive	0.00	Negative
**102**	L66	Stainless steel lancet	Negative	Negative	Negative	0.00	Positive	0.00	Negative

DME: Direct Microscopic Examination; Ct: Cycle of threshold
